# Supplementary material for: Are carbon emissions trading and green financial instruments synergistic? -Comprehensive quantitative research based on content analysis
Source: PLoS One. 2024 Mar 7;19(3):e0298601. doi: 10.1371/journal.pone.0298601 (PMC10919601; doi:10.1371/journal.pone.0298601)
Supplement: S1 Data — (ZIP) [file pone.0298601.s001.zip › Data/地级市Z_F工作报告.zip!/╜¡╬≈/╙Ñ╠╢╩╨/└┤╫╘╣1⁄2╓┌║┼í╛┬φ┐╦╩2╛▌═°í┐_2007.docx]

各位代表：

现在，我代表市人民政府，向大会作政府工作报告，请予审议，并请市政协各位委员和列席同志提出意见。

一、２００４年以来政府工作的回顾

近三年来，在省委、省政府和市委的正确领导下，全市人民以邓小平理论和“三个代表”重要思想为指导，全面贯彻党的十六大和十六届四中、五中、六中全会精神，以科学发展观统领全局，按照建设社会主义和谐社会的要求，认真落实国家和省一系列重大决策和部署，围绕建设和谐平安鹰潭和小康社会的目标，解放思想，敢闯新路，扎实工作，开拓进取，全市经济建设和各项社会事业跨入了一个新的发展阶段。

——经济总量迈上了新台阶。全市生产总值由２００３年的８５亿元增加到２００６年的１４０亿元（预计，下同），年均递增１８．２％。按现行汇率换算，人均生产总值达到１６００美元。财政总收入由８．６亿元增加到１９．１９亿元，年均递增３０．６％，增幅连年位居全省前列；其中地方财政收入由４．５亿元增加到８．３亿元，年均递增２２．８％。特别是县域经济实力迅速增强。２００５年贵溪市财政总收入在全省县（市、区）中率先突破１０亿元，预计今年财政总收入可达１４．５亿元。继去年余江县财政总收入超过１亿元，今年月湖区财政总收入也超过１亿元。

——经济结构调整取得了新进展。三次产业结构由１６．９４４．５３８．６调整到１２．８５６．２３１．０，三年内二产上升１１．７ 个百分点。工业成为推动经济快速增长的主导力量，农业基础地位更加巩固，现代服务业有了新的发展，个私经济占全市生产总值比重有了新的提高，多种经济成分共同发展的格局基本形成。

——经济发展增添了新后劲。全社会固定资产投资由３０．９亿元增加到７３．１亿元，年均递增３３．２％。社会消费品零售总额由２４．９亿元增加到４１．５亿元，年均递增１８．６％。

——人民生活水平有了新提高。全市城镇居民人均可支配收入由７０１４元增加到９８００元，年均递增１１．７％。农民人均纯收入由２７１７元增加到３９６８元，年均递增１３．４％。

近三年来，我们着力推进了八个方面的工作：

第一，坚持巩固农业基础地位农业农村经济发展较快。近三年来，我们始终高度重视“三农”工作。先后开展了千名干部进村宣讲一号文件、为民办实事和“种粮增收一百元”等一系列活动，抵御了洪水、干旱和禽流感等多种灾害，落实了国家和省一系列促进农业和农村发展的支农惠农政策。全市农民从政策性补贴和减免农业税中得到实惠共计２．９７亿元，平均每个农户约１５００元。粮食生产连年丰收，农民人均纯收入预计仍超全省平均水平。社会主义新农村建设开局良好，按照“五新一好”的目标，以“六改四普及”为切入点，共投入资金２６２０万元，建成了一批具有一定特色的示范点，广大农民群众初步感受到了新农村建设带来的成果。农业产业化进程加快，形成了一批农业产业化龙头企业，规模以上农业产业化龙头企业销售收入由６．４亿元增加到１６．３亿元，增长１．５倍。全市共实施农业项目２００多个，争取中央、省支农资金６亿多元。初步构建了以贵北早熟梨为主的果业产业区、贵南高效笋竹产业区、余江瘦肉型生猪产业区、洪五湖苗木花卉产业区、龙虎山天师板栗产业区５个产业区。全市农业增加值由１２．４亿元增加到１７．５亿元，增长４１．４％。

第二，坚持走新型工业化道路，工业化进程加速。近三年来，我们始终坚持把壮大工业总量与调整产业结构结合起来，注重转变经济增长方式，培育特色优势产业，工业主导型经济增长格局已基本形成。全市工业园区由小到大，已经成为吸纳就业的主渠道、招商引资的新平台、带动经济快速发展的增长极。现已建成面积达１３．２４平方公里，投入基础设施建设资金１１．７亿元，入园企业２６１家，其中已投产企业１４７家，提供就业岗位２．３万个，实现销售收入８５亿元，利税总额６．５亿元。优势企业迅速成长，江铜集团贵冶四期３０万吨铜冶炼技改项目和年产１０万吨阳极铜项目正在紧张建设，预计明年７月份竣工投产。贵电二期两台３０万千瓦机组成功投产，年新增税收约８０００万元。通过招商引资，先后引进了投资规模超亿元的骨干企业１８家，其中世纪阳光、中旺食品、梦娜袜业都是全国同行业的龙头企业。全市规模以上工业增加值由２７．５亿元增加到８２亿元，增长１．９８倍。产品销售收入由１３５．４亿元增加到４００亿元，增长１．９倍。实现利税总额由８．７亿元增加到８２亿元，增长８．７倍。

第三，坚持发挥旅游带动效应第三产业发展活跃。近三年来，我们围绕打造旅游品牌，做旺人气，开展了一系列旅游宣传促销活动，取得了较好效果。龙虎山景区入选全国首批国家自然与文化双遗产预备名录、被评为“中国最值得外国人去的５０个地方”和“江西十大最佳景区”，已经形成了具有全国影响的旅游品牌。成功举办了中国（龙虎山）道教文化旅游节、首届海峡两岸道教文化论坛等一系列旅游促销活动，拓展了旅游市场。２００６年，全市接待国内游客５８０万人次，年均递增１７．４％；接待境外游客４．２万人次，年均递增１７．５％；旅游总收入１４亿元，年均递增３４．１％；旅游创汇６２０万美元，年均递增２１．８％。个体私营经济不断壮大，房地产市场逐步规范。金融、保险、邮政、电信等行业取得长足进步，住宿、餐饮、文化娱乐等服务业健康发展。

第四，坚持以生态宜居城市为建设方向，城市化进程加快。近三年来，我们坚持按照“高起点规划、高标准设计、高质量建设、高水平管理、高效益经营”的要求，着力打造初具现代气息的新型城市。编制了《鹰潭市市域城镇体系规划》及其它一批专项规划，完成了江南水厂扩建、梅园大道综合改造等一批城市基础设施。鹰潭公园、东湖公园二期、垃圾填埋场、管道燃气一期、污水处理一期、胜利休闲广场、市体育中心、市人民医院住院大楼及门诊医技楼、市劳动力市场等重点工程正在抓紧建设。加强和改善了城市管理。集中整治城市规划区违法建筑取得阶段性成效。连续多年开展“告别陋习，树立新风”活动，市民素质进一步提高。城市环境卫生、交通秩序明显好转，物业管理进一步规范有序，服务质量明显改观。通过近三年的工作，城市框架不断拉大，城市功能不断完善，城市品味不断提升，我市先后被评为“中国优秀旅游城市”、“中国旅游竞争力百强城市”、“江西省卫生城市”。创建省级园林城市已通过省考核验收。

第五，坚持投资与消费双向驱动，经济发展实现了良性循环。近三年来，我们认真实施国家宏观调控政策，在促进固定资产投资增长的同时，努力扩大消费需求。全市共完成全社会固定资产投资１７７．１亿元，实施了交通、电力、水利等一批涉及发展大计和民生民利的重点建设工程。浙赣铁路电气化改造鹰潭段顺利完工，黄金埠电厂铁路专用线已建成，３２０国道鹰潭至余江段、２０６国道锦江至龙虎山段改造全面完成，龙虎山龚资线、贵溪文双线、彭上线、余江邓山线等一批公路建成通车。完成农村公路投资３．６亿元，改造农村水泥路８３０公里，全市１００％的乡镇通了水泥路（油路），９９．５％的行政村通了公路。贵溪铜城２２０千伏、罗河１１０千伏和龙虎山１１０千伏变电站建成投产，余江洪五湖１１０千伏变电站、黄金埠电厂锦江５００千伏开关站即将建成。完成水利基础设施建设投资１．５亿元，新建和加固堤防６．４公里，完成病险水库除险加固７７座。与此同时，高度重视扩大消费需求，着力改善消费环境，大力整顿和规范市场经济秩序，努力开拓城乡消费市场，全市社会消费品零售总额增幅逐年上升。

第六，坚持推进改革开放，经济社会发展活力增强。近三年来，我们按照完善社会主义市场经济体制的要求，深入推进了各项改革。深入贯彻《行政许可法》，切实转变政府职能，取消行政审批７６５项，深入开展政府机构改革，科学合理地设置政府机构，政府依法行政和决策科学化、民主化水平明显提高。推行并完善了行政事业单位新进人员面向社会公开招聘制。农村税费改革不断深入，全面取消了农业税。林业产权制度改革深入开展，水利产权制度改革平稳推进。粮食流通体制和市场化改革步伐加快，粮食市场进一步规范。企业产权制度改革遗留问题逐步得到解决。开展了部门预算制度试点工作，政府采购制度日趋规范。投资环境进一步优化。相继开展了“重大项目招商年”、“诚信优质服务年”和“安商亲商服务年”活动，健全了安商服务体系，完善了政务环境评议评价和涉企检查告知等制度，实现了由全民招商向“专业招商、全民安商”的转变，我市连续六年成为全省客商零投诉城市，被评为“２００５＇浙商最佳投资城市”。２００６年，全市实际引进内资预计可达２６．８亿元，其中５０００万元以上省外工业项目资金２３．６亿元；实际利用外资预计可达８０６４万美元、外贸出口预计可达４２５０万美元，分别增长２．０倍和１．０倍。

第七，坚持经济社会统筹发展，各项社会事业全面进步。近三年来，我们始终把发展社会事业放在更加重要的位置，促进经济建设与社会事业协调发展。科技事业取得新进展。我市先后被评为 “全国科技进步示范市”、“全省制造业信息化示范市”，被确定为“国家新材料产业化示范基地”。教育工作成绩显著。一批优质高中建设工程进展顺利，完成了农村现代远程教育工程。修建、改造中小学校舍１６．２１万平方米，基本消灭了现存一类危房。鹰潭一中两名学生获国际奥林匹克竞赛金奖，为我省、我市争得了荣誉。新型农村合作医疗试点全面铺开，农民看病难、看病贵的问题得到有效缓解。全国血防工作会议在我市余江县成功召开。环境保护工作取得实效，鹰潭空气质量和信江鹰潭段水质良好，分别达到国家二级和二类标准。严格执行国家土地政策，土地保护和利用更加规范。计划生育工作继续加强，新生婴儿性别比失调综合整治工作取得初步成效，人口自然增长率控制在省下达目标之内。文化市场日趋繁荣，农村广播电视“村村通”覆盖面进一步扩大。积极组团参加了江西省第十二届运动会，取得了金牌翻番、位次前移的好成绩，申办２０１０年省第十三届运动会取得成功。审计、统计、档案、新闻出版、人防、民族宗教、外事侨务、气象服务、防灾减灾、民兵预备役、老龄、残疾人等各项工作取得新进步。

第八，坚持以人为本，社会和谐稳定。近三年来，我们始终把实现好、维护好、发展好最广大人民群众的根本利益作为一切工作的出发点和落脚点，建立了困难群体的生活保障机制、社会矛盾的排查调处机制、突发性公共事件的应急处理机制。实施了积极的就业和再就业政策。积极扶持下岗失业人员自主创业，城镇登记失业率连续三年控制在３．８％以内，累计发放小额担保贷款９７００万元。社会保障能力进一步增强。提高了全市企业离退休人员养老金和城市居民最低生活保障标准，确保了国有企业下岗职工基本生活费和企业离退休人员基本养老金按时足额发放。实施了城市困难群众大病医疗救助，初步建立了农村最低生活保障制度。医疗保险制度进一步完善，出台了《鹰潭市市区城镇职工基本医疗保险实施办法》、《鹰潭市市直已改制国有企业退休人员参加基本医疗保险办法》和《鹰潭市市区城镇灵活就业人员参加基本医疗保险办法》。积极开展农民工工资清欠工作，较好地维护了农民工和被征地农民以及城市拆迁居民的合法权益。城乡市场繁荣、购销两旺，社会购买力和居民消费水平逐年提高。精神文明建设取得较好成果。开展了“建设和谐平安鹰潭，共创富民兴市大业”主题教育、“文明村镇创建”等多种精神文明创建活动，涌现出一批先进单位和先进个人。余江县九亭村被授予 “全国民主法治示范村”，施华山同志被授予 “全国公安系统一级英雄模范”荣誉称号。社会治安综合治理和安全生产工作得到加强。集中开展了一系列“严打”整治斗争，强化了社会治安防控体系建设。认真贯彻《信访条例》，依法解决群众反映的热点难点问题。出台了一系列突发公共事件应急预案，加强了生产安全、交通安全、防火安全和食品药品安全等各项安全工作，有效预防和减少了一般伤亡事故，杜绝了重特大安全责任事故的发生，我市连续３年获得“全省社会治安综合治理目标管理先进市”称号。

各位代表，近三年来，我们围绕建设社会主义和谐社会的宏伟目标，实现了全市经济社会发展新跨越。经过发展和探索，我们不仅积蓄了加快发展的物质基础，初步探索出了一条既适应时代要求又符合鹰潭实际的新的发展路子。更为重要的是，广大干部群众的思想观念和精神面貌发生了深刻变化，解放思想闯新路、众志成城促崛起的信心更加坚定，聚精会神搞建设、一心一意谋发展的氛围更加浓厚，全市上下焕发出蓬勃的生机和活力。所有这些，充分展示了富民强市的美好前景。这些成绩的取得，是省委、省政府和市委正确领导的结果，是人大依法监督、政协民主监督的结果，是各级政府、各部门、各单位辛勤劳动的结果，是驻鹰单位、人民解放军、武警官兵、离退休老同志及全市人民共同努力和大力支持的结果。特别需要指出的是，人大代表、政协委员及各民主党派、工商联、无党派人士、社会团体为推进我市经济社会发展发挥了重要作用。在此，我谨代表市人民政府，向全市人民，向所有关心、支持我市改革与发展的同志们、朋友们，表示衷心的感谢并致以崇高的敬意！

回顾近三年的工作，我们也清醒地看到，经济社会生活中还存在不少困难和问题，主要是：全市经济总量仍然偏小，区位和资源优势有待进一步挖掘，人民群众关心的热点难点问题还没有得到根本解决，新农村建设任务还很繁重，维护社会稳定还有大量工作要做，政府的办事效率还有待进一步提高。这些困难和问题，我们将高度重视，并坚持用改革和发展的办法，努力加以克服和解决。

二、今后五年的奋斗目标和主要任务

各位代表，市第六次党代会绘就了今后五年的宏伟蓝图。新一届政府将勇敢地肩负起历史重任，牢牢把握机遇，从容应对挑战，迎难而上，开拓进取，加快发展，加速鹰潭崛起步伐。今后五年，政府工作的总体思路是：坚持以邓小平理论和“三个代表”重要思想为指导，全面落实科学发展观，进一步加快改革开放和自主创新，进一步加速产业集聚和结构调整，进一步加强基础设施建设和完善城市功能，做强工业、做优农业、做旺旅游、做活商贸，不断提升城市辐射能力和新农村建设水平，不断提升和谐平安和小康社会构建水平，不断提升政府依法行政和公共管理水平，推进经济社会新发展，实现富民强市新跨越。

今后五年主要奋斗目标是：

——经济发展再上新台阶。到２０１１年，全市生产总值达到３００亿元，比２００６年增长１１４．３％，二、三产业比重分别达到５９．１％和３２．４％。财政总收入４０亿元，增长１０８．４％，其中地方财政收入１５亿元，增长８０．７％。城镇居民人均可支配收入和农民人均纯收入分别达到１５０００元和６０００元，年均分别递增９％和８．６％。

——城乡建设再求新突破。到２０１１年，城市化率达到４８％，主城区城镇人口达２７万人。新农村建设取得阶段性成果，三分之一以上村庄基本达到“五新一好”新农村标准。

——社会事业再现新繁荣。争取创办鹰潭本科学院，在农村全面实行免费义务教育，全面推行新型农村合作医疗，举全市之力办好省第十三届运动会。人口自然增长率、城镇登记失业率低于全省控制标准，社会保障面覆盖所有城乡。万元生产总值能耗年均下降４％。

实现今后五年的奋斗目标和主要任务，开创经济社会发展的崭新局面，要求我们必须明确主攻方向，突出工作重点，牢牢把握加快发展的主动权。

１、全力推进工业强市，决战工业１０００亿

坚持走新型工业化道路，以工业园区为平台，以壮大特色产业集群、扶持优势企业为重点，以机制创新、技术创新为动力，拓展发展载体，壮大产业集群，完善服务体系，全面提升工业化水平。力争２０１１年，全市工业产品销售收入突破１０００亿元，实现利税超过１００亿元。为实现这一目标，一要集中办好工业园区。通过推进工业园区土地集约化经营、产业链式化延伸、项目集群式组合、资源循环式利用，提高工业园区投入强度和产出水平。以鹰潭、贵溪、余江三个省级工业园区为支点，加快建设鹰潭３２０国道工业走廊，使之成为推进工业化的重要载体、招商引资的主要平台，在增强实力、集聚能量、辐射带动方面发挥重要作用。二要壮大特色产业集群。铜产业在今后较长时期是支持我市经济发展的重要力量，突出优势，加快扩张，做大规模，提升配套，延伸产业链，推进深加工，既能为鹰潭工业形成持续增长的骨架，也能培育新的经济增长点。为此，要以建设国家新材料产业化基地为契机，以江铜集团公司为依托，围绕铜冶炼能力的扩大，按照上下延伸、双向扩张的发展思路，延伸产业链，着力发展铜产业集群，力争５年内阴极铜产能达９０万吨，铜材加工能力达１００万吨，铜材加工产品销售收入达６００亿元，把鹰潭打造成为国内最大、世界有重要影响力的铜产业基地及全国最大的铜产品集散中心。借“眼镜之乡”金字招牌，努力在眼镜生产成龙配套上下功夫，着力发展眼镜产业集群，力争５年内全市眼镜专业园区落户企业１００家以上，实现年产值１５亿元以上，把鹰潭打造成为全省乃至全国有重要影响力的眼镜产业基地。围绕发展钟表、仪器、仪表、电子通讯、精密光学等系列产品，着力发展微型元件产业集群 用５年时间使其年销售收入过３亿元，从业人员达７０００人，把鹰潭打造成为在全国有重要影响力的微型元件生产制造基地。三要扶持优势企业。精心扶持水表、节能灯、医药、化工、食品、纺织等优势企业发展壮大形成核心竞争力，扩大市场占有率，力争用５年的时间实现全市重点优势企业达５０家，其中年销售收入过５亿元的企业１０家，１０亿元以上的企业５家，超２０亿元的企业２家。四要实施品牌战略。以“名牌兴企”的发展理念，帮助企业制定品牌战略目标体系，鼓励并帮助企业创全国、全省知名企业、名牌产品，努力走出一条以产品带企业、以企业带行业的链式发展之路。五要完善企业发展服务体系。在服务驻市企业方面，由过去单纯注重外部环境的服务方式转变为更加注重同心同向谋发展、互动合作促发展的战略联盟方式上来，以谋求规划、思路上的对接和企业发展趋势的共识，建立双方高位推动的领导机制、重大项目协调推进机制，营造良好政策环境和资源、功能共享的城市发展环境。建设全国铜冶炼及加工工程技术中心、全国铜产业技术人才培训基地、中小企业信用担保中心，并在电力供应、融资信贷、税收政策、用工培训等方面提供全方位的服务和支持。

２、稳步发展农村经济，力争社会主义新农村建设走在全省前列

按照“生产发展、生活宽裕、乡风文明、村容整洁、管理民主”的总体要求，以“五新一好”为目标，发挥政府主导、农民主体、全社会参与作用，坚持走以城带乡、以工促农、城乡共同发展的新路子，推动社会主义新农村建设不断取得实效。一要以发展生产为根本，做强优势特色产业。按照“适应市场、特色兴业、区域布局”的要求，做好“山、水、田、畜、工”五篇文章，实现优势农产品的集中布局，推进规模经营，在稳定粮食生产的基础上，不断优化农业产业结构，促进区域块状经济加快发展。重点打造以贵北早熟梨为主的果业产业区、贵南高效笋竹产业区、余江瘦肉型生猪产业区、洪五湖苗木花卉产业区、龙虎山天师板栗产业区，重点做大刘垦花生市场，整合完善大米加工市场。力争５年内全市经济作物产值占种植业产值的比重、养殖业产值占农业总产值的比重均达到６５％以上。围绕优势产业，着力发展农副产品加工、储藏、保鲜、运销业，加快推进农业产业化进程。力争用５年的时间培植２－３个科技含量高、带动力强、销售收入达５亿元以上的龙头企业，全市规模以上农业产业化龙头企业实现销售收入突破３０亿元。二要以强化农业基础设施建设为前提，提高农业综合生产能力。加快万亩以上圩堤除险加固工程建设，完成市区城市防洪日元贷款项目，全面完成全市小（二）型以上病险水库除险加固工程，基本完成信江、白塔河、罗塘河等重点河段河道整治工程，主要防洪保护区堤防及城市防洪建设达到规划确定的防洪标准。加大万亩以上灌区节水改造力度，完成余江县白塔渠水利血防工程，全面解决全市农村人口饮水安全问题，进一步改善农村生产生活用水条件。三要以环境整治为切入点，改善农民生活环境。坚持以规划为龙头，因地制宜搞好村镇体系规划建设，从农民最关心、最迫切需要解决而又能做到的现实问题入手，整治村容村貌，着力抓好“六改四普及”，加快村庄和庭院绿化、美化，逐步完善农村道路、水电、通讯等公共服务设施，全面改善农村人居环境。５年内对１２００个左右的自然村进行集中整治，尽快实现农村面貌的整体改善。四要以培养新型农民为目标，提高农村民主化管理水平。深入开展“文明村镇”创建和道德、法律、文化、科普“四进农家”活动，培养有文化、守纪律、懂技术、会经营的新型农民。完善村民自治，规范“一事一议”，推进村务、政务公开和民主管理，提高农民群众自我教育、自我服务、自我管理的水平。

３、着力推动旅游兴市，全面提升第三产业水平

充分挖掘“大工业、大交通、大旅游”的区位优势和发展潜力，加快改造和提升传统服务业，大力发展现代服务业。一要做大做旺旅游业，打造世界知名、国内一流的旅游品牌。坚持政府主导型旅游发展战略，按照大旅游、大开发、大市场、大品牌的发展思路，加快实现从旅游资源大市向旅游经济强市的跨越。积极推进龙虎山申报世界“双遗产”， 争创“全国５Ａ级景区”。加快开发新型特色旅游项目，突出“道教文化、崖墓探奇、绿色生态、保健养生、铜都工业、地质科考、商务休闲”七大旅游主题。科学配置旅游线路，拓展道教文化内涵，延伸旅游产业链，完善旅游服务功能，打造旅游城市新形象，实现旅游产品由单一观光型向综合型转变，产品规模达到３—４日游的水平，重点推出２—３日游线路，形成“淡季不淡，旺季更旺，四季皆可游”的旅游旺势。把鹰潭建设成为中国道教文化圣地和“长珠闽”地区重要的多功能复合性旅游目的地，努力创建“中国最佳旅游城市”，力争实现旅游总收入占全市生产总值１５％的目标，使旅游业成为充满活力的优势产业、富民产业。二要以“好口子”综合优势为取向，把鹰潭建设成江西重要的现代物流中心。抓住交通部将我市列入国家道路运输二级枢纽规划的机遇，按照政府引导、市场运作、企业为主、分步实施、逐步推进的原则，从建立物流园、公共物流信息平台、引优扶强物流企业入手，重点引进一批有实力、有潜力的物流企业，重点扶持一批现有骨干物流企业，充分整合物流资源，逐步壮大物流产业，降低物流成本，使我市交通优势更加凸显，投资环境更加完善。三要大力发展个私经济，提高个私经济在经济总量中的比重。按照“巩固存量、挖掘潜力、扩大总量，放活增量、提升质量”的思路，以全民创业为契机，毫不动摇地鼓励、支持和引导个私等非公有制经济加快发展，切实落实各项扶持政策，放宽市场准入，进一步拓宽发展领域和空间，重点支持个私企业大力发展旅游、商贸流通、运输、餐饮服务等传统第三产业。同时，鼓励发展现代金融保险业、信息服务业，有序发展会计、律师、公正、资产评估和信用担保等新兴服务业，增强综合竞争力，繁荣做活第三产业。

４、加速市县一体化进程，构筑大鹰潭城市发展格局

按照“一个核心区、三个特色区”的构想，积极推进“四区合一”进程，构建“半小时经济圈”。通过“东连、西接、南扩、北拓”，拉开城市框架，力争用５年时间实现主城区建成区面积３０平方公里、主城区城镇人口规模２７万、全市城市化率４８％的目标。为此，一要立足高起点，认真做好城市规划。抓紧做好《鹰潭市城市总体规划》、《鹰潭市主城区近期建设规划》、《夏埠区控制性详细规划》的报批工作，编制《鹰东区控制性详细规划》，做好城市景观规划设计、城市地下空间开发等规划工作，以规划引导建设。二要追求高质量，切实搞好城市建设。修建鹰潭市区至贵溪的城际快速干道，完成鹰潭市区至贵溪的３２０国道拓宽改造工程，以形成环状路网，为鹰潭市区与贵溪城区一体化创造条件。强化基础设施建设，不断完善城市功能。完成胜利西路、龙虎山大道、四海路延伸工程，启动夏埠沿江大道建设，改造站江路、环城东路和府前路，加快城市供气、供电、供水、环保设施建设，完成管道燃气一期工程、污水处理一期工程建设，完善城市的旅游配套服务功能。改造完善老城区，做精做美梅园区，做热做旺高桥区，启动开发夏埠区，加快建设宝山片区，建设一批环境优美的高品位商住小区。完成东湖公园、滨江公园、胜利休闲广场工程，将鹰潭公园改造成为集市民休闲、娱乐、健身为一体的江西乃至江南最具特色的城市公园。着力推进城乡一体化进程。与城市发展相呼应，努力构建“一带两轴三片区”的重点城镇体系。三要坚持高水平，大力加强城市管理。建立健全城乡规划督察员制度，开展城乡规划效能监察。理顺城市管理体制，积极探索城市管理综合执法，建立城市管理长效运行机制。继续依法整治城市规划区违法建筑和非法营运、占道为市等影响市容市貌的不良现象和行为加强城市环卫保洁、城市绿化、城市交通管理工作，努力营造管理有序、秩序良好、环境优美、安全文明的宜居城市环境 。

５、以改革开放为强动力，着力提升开放型经济发展水平

坚持用改革的办法破解难题，用开放的成果激发活力。通过不断深化改革、扩大开放，破除一切束缚生产力发展的体制机制障碍。

坚定不移地推进和深化各项改革。进一步推进投资体制改革，规范投资项目的审批、核准和备案；继续抓紧解决国有改制企业遗留的债务和资产变现等问题；积极推进行政管理体制改革，改善运行机制和管理方式，提高办事效率；深化以乡镇机构、农村义务教育和县乡财政体制改革为主要内容的农村综合改革，推进和完善林业产权制度、水利管理体制、土地征收征用制度等项改革，积极化解乡村债务，防止农民负担反弹；妥善解决国有农场改革遗留问题。

坚定不移地实施大开放主战略。坚持以招商引资为主抓手，瞄准重点区域，注重产业选择，招大引强，力争５年内实现引进省外单项投资５０００万以上的工业项目实际投资额、利用外资总量和外贸出口总额分别比前五年翻一番。坚持巩固优化投资环境，增强安商意识，强化安商责任，完善安商制度，创造良好的政务环境，降低投资商务成本。加快口岸建设，力争五年内在鹰潭设立海关分支机构和进出口检验检疫分支机构，为外贸出口提供优质便捷的服务。

坚持招商引资同全民创业相结合，把推动全民创业作为加快富民强市、实现加速崛起的重大举措，在激励创业、扶持创业、成就创业上取得新成果，为鹰潭经济社会持续快速健康发展增添后劲。

６、以做实项目为支撑，夯实经济社会发展基础

坚持“项目带动”战略，充分发挥重大项目建设对经济社会发展的促进和拉动作用。今后五年，我市将实施涉及工业、农业、商贸、旅游、交通、能源、水利、生态等１０大类４８９项规划支撑项目，其中重大建设项目３５２项。抓好这些建设项目的实施，对于加快我市经济社会发展、增强城市综合配套能力和发展后劲具有重大意义。为此，要进一步完善项目策划、协调服务和推进机制，在项目立项、用工管理、资金筹措、税费收缴等方面为项目建设提供支持，努力提高项目实施的成功率。要精心调度，落实项目建设责任制，解决项目建设中的实际问题，促进项目早日开工、建设和投产见效。

坚持统筹规划、合理布局、量力而行、适度超前的原则，进一步加大投资力度，推进交通、电力、信息基础设施建设，使基础设施保障能力与经济发展水平相适应。

建设快速便捷的综合交通体系。积极配合建设景鹰高速公路和鹰瑞高速公路，同步建成景鹰高速鹰潭连接线和鹰瑞高速龙虎山连接线。加快县乡公路改造和通村公路建设，逐步形成与高速公路主骨架互通和配套的公路网，实现市至县通一级公路，县至乡通三级以上公路，１００％行政村通等级水泥路，５０％的自然村通水泥路。在已争得南昌至上海城际快速列车停靠鹰潭的基础上，支持铁路鹰潭车站尽快实施配套改造工程，实现鹰潭４小时到达上海。新建鹰潭港客运码头和作业区，进一步强化鹰潭交通枢纽地位。

建设安全可靠的能源保障体系。加强电源点和供电网建设，完成贵溪电厂两台６０万千瓦“以大代小”改造工程，对接毗邻的黄金埠电厂建设，将鹰潭建设成为江西重要电力供应基地。加快输变电工程建设，到２０１１年，拥有３５千伏变电站１７座，１１０千伏变电站１６座，２２０千伏变电站４座，５００千伏变电站１座，使全市城乡都能享受到充足的电力保障。

加快信息化进程。加快信息技术在经济社会领域的普及应用，提高经济社会运行质量和效率。实行重点突破、以点带面、全面推进，加速政务信息化、企业信息化、公共服务信息化、农业农村信息化。

７、大力实施“科教兴市”战略，切实推进各项社会事业

全面落实科技是第一生产力和人才是第一资源的战略思想，全面繁荣各项社会事业，形成比较完善的现代国民教育体系、科技和文化创新体系、全民健身和医疗卫生体系，不断提高人民群众的思想道德素质、科学文化素质和健康素质，促进社会主义物质文明、政治文明和精神文明协调发展。

提升科技创新水平。坚持“自主创新、重点突破、支撑发展、引领未来”的方针，加快科技进步示范市建设，建立健全科技服务体系、区域创新体系和技术创新体系，争取在生物医药、水表、新材料、节能灯等重点领域取得一批自主知识产权，争取在农业、资源、生态环境等领域取得一批重大科技攻关成果。到２０１１年，力争科技进步对经济增长的贡献率达５０％以上。

优先发展教育事业。重点加大农村教育投入，全面推进素质教育，促进各级各类教育和城乡教育协调发展。高水平普及九年义务教育，全面实施免学杂费和贫困生免教科书费、补助寄宿生生活费政策，并提高公用经费标准。大力发展职业教育，积极发展高等教育，争取创办鹰潭本科院校。鼓励和支持社会力量办学，大力促进民办教育。

积极发展文化事业。力争２０１１年各县（市、区）建成功能齐备的文化设施，各乡镇都有综合文化站，行政村有文化活动室。筹建鹰潭市艺术中心。做好角山文化遗存申报国家文物保护单位工作。大力推进广播电视数字化及相关技术改造，扩大农村广播电视覆盖率，基本实现村村通。扩大文化对外交流，发展新闻出版业，创造更多更好适应人民群众需求的优秀文化产品，充实人民群众的精神生活。

全面加强卫生事业。积极推进医疗卫生体制改革，调整和优化医疗卫生资源配置，增加公共卫生支出，建成市人民医院住院大楼及门诊医技楼。进一步完善疾病预防控制体系、突发公共卫生事件医疗救治体系、突发公共卫生事件应急体系和卫生执法监督体系，不断提高公共卫生应急能力和防病治病能力，切实保障人民群众的身体健康。按照“低水平、广覆盖”的要求，突出大病统筹和对困难群体的医疗救助，争取用五年左右的时间，建立覆盖城乡的医疗保障体系。进一步扩大新型农村合作医疗试点，建立城镇居民合作医疗制度，切实解决好群众看病难、看病贵的问题。

大力推进体育事业。以成功申办省第十三届运动会为契机，掀起全市发展体育事业新高潮。高质量完成市体育中心建设，尽快建成市青少年活动中心，加快社区及中小学校体育设施建设步伐，尽快改变体育设施落后的状况。不辱使命，不负重托，举全市之力精心筹办好江西省第十三届运动会。

加强人才队伍建设。抓住培养、吸引、用好人才三个环节，强化党政人才、企业经营管理人才、专业技术人才三支队伍建设。大力实施人才集聚、人才开发和领军人才建设工程，建立和完善有利于人才成长和优秀人才脱颖而出的激励机制，积极营造人才“引得进、留得住、用得好、流得动”的良好环境。

８、坚持以人为本的执政理念，合力构建和谐小康社会

坚持以人为本，高度关注民生，从人民群众最关心、最直接、最现实的利益出发，将新增财力进一步向困难群众倾斜、向农村倾斜、向基层倾斜、向社会事业薄弱环节倾斜，让广大人民群众共享改革发展的成果。

千方百计扩大就业和促进再就业。继续大力实施积极的就业政策，全面落实和完善健全促进就业再就业的各项优惠政策。进一步拓宽就业门路，努力增加就业岗位。加大就业技能培训力度，突出解决好农村富余劳动力就业问题。积极鼓励自主就业，推动全民创业，进一步完善劳动者自主择业、市场调节就业和政府促进就业的长效机制。加快劳务输出步伐，缓解就业矛盾。力争到２０１１年，全市新增城镇就业１２万人，城镇登记失业率控制在４％以内。

千方百计保障城乡居民基本生活。继续完善城镇企业职工基本养老保险制度，积极探索和大力实施失地农民养老保险。深化基本医疗保险制度改革，积极帮助困难企业和改制企业职工参加医疗保险。同时，大力加强城镇职工失业、工伤、生育保险制度建设。按照“应保尽保，动态管理”的原则，建立健全覆盖城乡的最低生活保障体系，并逐步增加保障经费，提高城乡居民生活最低保障能力。积极推进经济适用房和廉租房建设，５年内建造一定数量的经济适用房，让中低收入家庭能够买得起、租得到住房。

千方百计维护社会稳定。正确处理改革发展稳定的关系，妥善处理社会矛盾，适应社会结构和利益格局的发展变化，建立健全科学有效的利益协调机制、诉求表达机制、矛盾调处机制和权益保障机制。高度重视信访工作，认真做好人民内部矛盾排查调处工作。加强社会治安综合治理和法制宣传教育工作，推进基层和谐创建活动，完善“管防控打”一体化治安防控体系，依法严厉打击各种违法犯罪活动。加强国家安全工作。健全安全生产长效机制，强化企业安全生产和食品药品安全的自我约束和责任追究，完善高效运作的安全生产监管体系，全面提高安全生产水平。

千方百计保护生态环境。要通过五年的努力，力争打响绿色鹰潭、生态鹰潭品牌。进一步加强污染防治和污染排放总量控制，确保信江饮用水源和泸溪河旅游资源水质继续稳定在国家二类标准，大气质量继续稳定在国家二级标准。按照花园城市、园林城市和生态城市的要求，着力改善城市环境质量，为构建和谐社会提供良好的人居环境。积极开展生态公益林、江河湖库防护林、森林公园、自然保护区等生态工程建设，努力提高森林覆盖率。加强土地、资源的保护和管理，确保动植物、生物生态资源的多样性。大力发展循环经济，在全社会形成资源节约的增长方式和环境友好、健康文明的消费方式。

９、加强政府自身建设，推进政府管理创新

各位代表，新一届政府重任在肩，全市人民寄予厚望。为实现新一届政府提出的目标和任务，推进鹰潭更快更好地发展，带给群众更多更大的实惠，我们要以更加务实、更加敬业、更加规范、更加高效的新形象、新风貌，履行职责，不辱使命。为此，必须大力加强政府自身改革和建设。一要坚持将依法行政贯穿于政府工作全过程，以法律约束行政权利，靠制度规范行政行为，将政府工作纳入法治轨道，努力建设法治政府。二要严格落实行政决策责任制，抓紧完善行政执法评议考核机制，建立健全行政执法过错责任追究制度。加快建立以行政首长为重点的行政问责制，做到有错问责、无为问责，尤其是对损害人民群众利益的突出问题，严格依法追究责任，努力建设责任政府。三要坚持把实现好、维护好、发展好人民群众的根本利益作为一切工作的出发点和落脚点，进一步强化政府工作人员的服务意识，努力建设服务政府。四要牢固树立科学发展观和正确政绩观，大力弘扬求真务实的优良作风，说实话、办实事、求实效，增强大局意识和全局观念，创新工作方式和服务手段，不断提高行政效率，努力建设效能政府。五要坚持标本兼治、综合治理、惩防并举、注重预防的方针，以改革和制度建设为重点，继续推进惩治和预防腐败体系建设，努力建设廉洁政府。

各位代表！实现今后五年的各项目标，我们面临的任务繁重而艰巨，我们肩负的使命神圣而光荣。让我们高举邓小平理论和“三个代表”重要思想的伟大旗帜，紧密团结在以胡锦涛同志为总书记的党中央周围，在省委、省政府和新一届市委的领导下，团结奋进、开拓创新，为实现全面建设小康社会的宏伟目标，促进鹰潭加速崛起而努力奋斗。
